# A reappraisal of public engagement in Oxford during the pandemic: three case studies

**DOI:** 10.1186/s40900-022-00343-z

**Published:** 2022-03-28

**Authors:** Milly Farrell, Clare Wilkinson

**Affiliations:** 1 Wellcome Centre for Ethics and Humanities, University of Oxford, Oxford, UK; 2 Science Communication Unit, University of the West of England, Bristol, UK

**Keywords:** Public engagement, COVID-19, Pandemic, Inclusivity, Diversity

## Abstract

**Background:**

The COVID-19 pandemic has resulted in the majority of public engagement with research work moving online. This shift to online engagement is likely to affect inclusivity and diversity in such events and this requires further consideration as a result of the pandemic. Through comparing case-studies both pre-dating and during the pandemic, we are able to discern areas for ongoing improvement and learning in the public engagement sphere.

**Main body:**

The public engagement work of the Wellcome Centre for Ethics and Humanities has sought to include a broad discussion on its research from a range of demographics, attempting to be inclusive in the engagement work of the Centre. However such efforts have not always been successful and we reflect here on two different pre-pandemic ‘in-person’ case studies assessing public views on vaccination and medical data sharing. In contrast we compare these pre-pandemic activities to a fully online case study coordinated and completed during the pandemic. These three case studies are compared and assessed for evidence of their efficacy in a post-pandemic world.

**Conclusion:**

Research and public awareness benefit when multiple views are included in engagement events. Broader demographics enrich our ways of understanding societal responses to healthcare issues such as vaccination, data sharing and social responsibility. The move to online engagement as a result of the pandemic may open opportunities to widening engagement geographically, but it could also pose a threat to inclusivity with certain public groups on a more local level. Enabling access to online engagement is key, but considerations must be made regarding the new barriers created by a solely online world and the many groups of people inadvertently excluded from this work.

## Plain English summary

It is widely recognised that research is improved when public groups are informed or consulted in this work. In university research settings, academics are actively encouraged to engage the public and seek their input and opinion. However, in order for this work to be of benefit to all involved, the public groups included should be diverse and represent a wide range of society.

The Wellcome Centre for Ethics and Humanities is a Wellcome Trust funded research centre at the University of Oxford. The Centre aims to assess and interpret some of the major ethical questions of our time, essentially questioning what it means to be human in a world of rapid technological change. The public engagement work of the Centre strives to include public opinion on its research and raise awareness of the many ethical issues we face as a species.

Before the onset of the pandemic in March 2020, the Centre held a range of ‘in-person’ events, where public groups and researchers were able to communicate in the same space. However, since the pandemic, this engagement work has moved fully online. Through evaluating these activities, we have been able to interpret which groups of people have participated. Through capturing this information, the Centre can monitor both the reach of its work and whether diverse groups of people have been included. We have found that there are a range of barriers and complications involved in solely online engagement and that future online work must address these issues, should it continue.

## Background

The ongoing coronavirus pandemic has resulted in dramatic global shifts to the way we connect with one another. Alongside the pandemic entirely new terminology has developed to describe and define this disruption. One particular term, now in common pandemic vernacular in the UK, is that of the ‘bubble’; essentially defining an exclusive group of close friends, family or households in which individuals are allowed to circulate, to control the spread of the virus. However, the concept of ‘bubbles’ is also akin to ongoing work in public engagement with research. Universities and research institutes have faced criticisms that they are bounded by their ‘ivory towers’ for many years [2, 27] and have a tendency to ‘preach to the converted’ [18]. Indeed this is one of many drivers for broader research involvement. Despite efforts to reach a wider range of public, patient and service user perspectives, public engagement can also be critiqued for operating with its own ‘bubbles’, struggling to connect with groupings of people beyond its sphere, communities which are sometimes described as the ‘hard to reach’ or ‘under-served’ [28].

For public engagement professionals, there is a constant quest to be more inclusive and to seek a broader discourse on research that encompasses multiple demographics [8, 14] beyond those that are already interested or engaged, as well as a diversity of perspectives. Dawson’s [7] work on informal research engagement environments, such as science centres and museums, suggests most visitors tend to be from White ethnic backgrounds, younger, with children and from higher social classes. Her work has led to an increased focus on the disenfranchising of people from research communication and engagement on the bases of ethnicity, gender, low-income, and other protected characteristics, such as disability [7]. Whilst research in health communication has also pointed out the deficiencies in communication which can fail to be culturally tailored or respectful to potential audiences [5, 10, 15, 26]. Acknowledging these problems, a variety of emerging projects are seeking to ensure co-production, involving participants in design and implementation, in the development of public engagement and involvement [11, 12], including within the constraints of the pandemic [13, 19, 23]. And increasingly the research engagement sector is also reflecting on its own history and weaknesses, which may have excluded multiple demographics and therefore multiple perspectives [4, 9, 22, 24].

We believe that the current pandemic presents both opportunities and threats to breaking down such barriers to impactful engagement with research. Here we reflect on this issue by summarising three engagement projects at the Wellcome Centre for Ethics and Humanities, based at the University of Oxford. These activities present evidence of distinctive demographic shifts in recent engagement work in Oxford, both before the onset of the pandemic and during, recognising that a diversity in demographics is key to supporting a diversity of viewpoints. In considering these examples, we utilise Humm and Schrögel’s [14] recommendations for engaging underserved groups, including listening to them, reducing the distance, going where the people are, cooperating with stakeholders and multipliers, illustrating the relevance of research for daily life, as well as opening up and making visible the research process, and creating sustainable, long-term activities. Offering an overview of evaluation findings from these engagement projects, we will also assess the evidence presented for engagement activities in a post-pandemic world, essentially questioning how and if such activities would work and whether public engagement has the potential to become more inclusive as a result.

## Public engagement at the Wellcome Centre for Ethics and Humanities

The Wellcome Centre for Ethics and Humanities (WEH), based at the University of Oxford in the UK was established in 2017 to address ethical issues and challenges arising from novel scientific and technological interventions in global health. Public engagement is core to the work of the Centre, given that ethical issues and questions cannot be addressed without public input or consultation. The WEH researchers themselves are specialists in a range of fields including medical history, medical ethics, psychology, philosophy and mental health, with projects varying across these disciplines. As such, the public engagement strategy was developed to ensure that its aims met both the objectives of the research at WEH, but also remained inclusive and open to the key audiences that the Centre strives to incorporate in its engagement. From the outset of this work in 2019, the key audiences for WEH’s engagement programme were identified in the Centre’s public engagement strategy as local Oxfordshire residents, artists and arts communities, research participants and patient groups [25]. This strategy is revised annually with consultation around methods, aims and key audiences provided by the WEH’s Public Engagement Advisory Group. This groups consists of 10 members; five of whom who sit within the WEH (from a range of career levels) and four of whom are external to the WEH, with one advisor external to the University.

The medieval City of Oxford is the site of the oldest university in the English-speaking world, with evidence of teaching dating back to the late eleventh century [3]. The University is an assortment of historical colleges, museums, libraries and other buildings that have been interwoven into the fabric of Oxford’s urban design, and dominate the architectural landscape of the City. Since their medieval foundations, the City and the University, have been intrinsically linked. However, a segregation has existed between the historical sub-groups of ‘Town and Gown’ (the local public and the University) which holds a long legacy in Oxford’s history. Oxford is now a well-populated city with over 150,000 residents and a thriving industrial sector. The societal divide present in Oxford has been amplified by the dominance and wealth of the University over the centuries, whilst some areas of the city are known to have high levels of deprivation and low social mobility even in the present day. The University and the wider population of Oxford continue to address this division through a range of projects focused on,widening participation within the student body, EDI work, communications and public engagement activities, to involve broader demographics in all aspects of the institution. In connection to this work the city of Oxford is home to a variety of independent free public science and arts festivals and events.

The perceived exclusivity of the University however still persists in many aspects of its work and addressing this issue is an ongoing priority for the University. Public engagement at the Wellcome Centre for Ethics and Humanities was consolidated in 2019 and initially placed a focus on forging collaborative projects with local groups and staging primarily information based events at educational festivals. These events were evaluated for evidence of audience demographics, participant satisfaction, educational content and where applicable, evidence of any learnings or change in opinions. Inevitably all ‘face to face’ activities ceased with the onset of the pandemic in March 2020 and work has since moved to be exclusively online, with recent projects including online theatre events and a photography exhibition. Attempts were made in late 2020 to produce activities on a hyperlocal level, enabling ‘in person’ activities for a small local group to engage safely and in line with government restrictions. These plans were thwarted by the second UK national lockdown coming into force in November 2020, which resulted in the exhibition moving to a solely online format.

Ongoing public engagement plans in 2020 and 2021 largely focussed on online activities or at least had online contingency planning in place to facilitate blended events. Our concern is that such work will inevitably discriminate against public groups identified in WEH’s strategy as key audience members. These public groups include children and young people (who may have constraints in accessing devices from a home setting, potentially where multiple or other devices are required and in use), patients and service users (who may also have online access constraints and/or lack technological capabilities to engage) and health professionals (who may have time constraints during a pandemic). On a basic level this engagement is reliant on public access to both a digital device with a reliable internet connection and the ability to use this device and supporting technology, but also on the assumption that even if such access barriers are removed, there will be the desire, interest, energy or time to participate. Through reviewing two of WEH’s most recent ‘face-to-face’ events, the first a public debate on mandatory vaccination held in October 2019, and the second, a Citizen’s Jury on data sharing that took place in February 2020, alongside a mid-pandemic solely online activity, we reflect on the issues identified with inclusivity and diversity and what implications this may hold for an exclusively online or ‘blended’ (face-to-face and online) engagement sector.

## Case studies

### Case study one

In October 2019, WEH took part in the local Oxford ‘IF’ Science and Ideas Festival [21]. This local festival aims to particularly reach audiences of lower socio-economic status who have not previously been enabled to participate in the festival. The festival team evaluate any evidence of this reach year-on-year. One of the events organised by WEH as part of this festival, was titled ‘*In Our Blood: is it our social responsibility to vaccinate?*’ The aim of this event was to encourage public discussion of the ethical issues surrounding vaccination hesitancy and to establish whether the event led to any shifts in perspectives on the topic of mandatory vaccination, which is an ongoing research focus at the centre. The format was an open panel discussion, with a large part of the event spent taking questions from the floor. Panellists included a vaccination historian, vaccination scientist, social scientist specialising in vaccination hesitancy and a philosopher who provides arguments in support of mandatory/compulsory vaccination programmes.

The event was held at New Road Baptist Church in the centre of Oxford and had good attendance (n = 68). When questions were opened to the public, it became evident that a group of around 10 attendees had a range of concerns with the efficacy and safety of vaccination and identified themselves as ‘vaccine sceptics’. At times it was challenging for the chair to manage discussions as members of this group were clearly distressed by both their prior experiences and also appeared to be angered by some previous work of one academic on the panel. The chair’s diplomatic management of the event meant there was opportunity given for all to speak and any signs of angry rhetoric or personal attacks were politely handled.

Evaluation was conducted via short questionnaires left on each audience member’s chair, which were completed on the night and returned anonymously at the exit. Participants at the event were not asked to provide details of their gender, age or residency due to concerns regarding duplication with other evaluation that may have been happening as part of the Festival. The results of these questionnaires showed that broadening the discourse on this occasion proved to many attendees that the assumption that all attendees in Oxford are pro-vaccine was invalid. In fact the event showed that a multitude of viewpoints existed within the region and vaccine hesitancy was more prevalent than expected. Although the purpose of the event was not to advocate for mandatory vaccination, but to present a selection of viewpoints on the issue, evaluation indicated that some felt swayed by a mandatory vaccination argument, given the strong and assertive presence of vaccine sceptic audience members. With regards to shifting perspectives, 25% (n = 9/36) of respondents indicated that the event had changed their view on mandatory vaccination and highlighted the dangers of misinformation.

### Case study two

The second case study, took place in mid-February 2020 at the Oxford Town Hall, just prior to the onset of the pandemic in the UK. This engagement activity was a Citizens’ Jury titled *‘Debating Data: How should your health data be used or shared?’* and sought to include public opinion on the issues of health data use in research and in commercial settings. The premise of the one day event was to outline the differences and current uses of genomic, pathology and imaging data in the UK. A primary aim was to encourage public discussion on these topics and capture public ‘verdicts’ on how these health data groups could and should be approached in future research and work at WEH.

Unlike the previous case study of the vaccination event in 2019, which linked to an external festival, this Citizen Jury was organised and run internally, with no external partners involved in its coordination. This latter point may have affected the marketing and reach of this event, which resulted in public participants (n = 20) representing a relatively narrow demographic. Eight men and 12 women attended and the majority of participants were white. Age range was spread across the age-spectrum, with the largest cohort (n = 6) identifying as within the 60–70 years age bracket. 16 participants resided in Oxfordshire and four came from outside of the region. Some participants identified as being part of the existing University Patient and Public Involvement (PPI) network, which would suggest either a prior interest or knowledge and awareness of certain healthcare issues and topics. Our recruitment and marketing strategy for the event inevitably led to this linear demographic, which may have limited discussions around the issues presented.

In the case of this event, consensus was reached amongst all groups reporting back, with points raised including the need for more protection for genomic data, recommendations around consent processes, and the oversight of commercial uses. An online evaluation survey was conducted post-event, which was anonymously completed by 12 of the 20 public participants. Most participants (80%) said the event was enjoyable, and felt the event was well organised and the format of the day worked well (70%). In the case of this evaluation one participant questioned the event, and its intentions, but there were also comments that the event would have benefited from a wider cross-section of participants, indicating that lack of representation is something participants are also conscious of.

### Case study three

The final case study presented here covers an engagement activity that was instigated during the pandemic and launched during the second UK lockdown in November 2020. Given the national situation and parameters created by the crisis, this activity was designed to be both a COVID-safe ‘in person’ event and also viewable online.

The project team began working on a photography exhibition titled *‘Indoors: experiences of older people during lockdown’* in early summer 2020, as a collaborative exhibition with a London based portrait photographer. As the UK emerged from its first lockdown and people began to interact throughout the summer, the team optimistically worked on an in-person exhibition of photographs that would accompany text panels written by WEH researchers. The four researchers involved in the project, had been investigating the various ethical and social issues created by pandemic restrictions. The initial plan was to install the exhibition inside the windows of a central London venue, with all images and text fully viewable from street-level. This approach would therefore create a socially-distanced and outdoor display, as a hyper-local activity in East London. The people photographed as part of the project were in communities local to exhibition, so WEH intended to advertise this in-person aspect of the project to local community groups. However, the identified audience widened out to older people in the UK, their neighbours, carers and relevant charities, who would inevitably be restricted in visiting this display. As a result of both the uncertain situation with the ongoing pandemic and a key audience being both widespread and vulnerable, the team also planned an online version of the exhibition.

As November 2020 approached it became apparent that a second national lockdown was looming. This meant that the team were unable to install the socially distanced in-person exhibition in London, and were restricted to this project launching and remaining entirely online. An evaluation survey link was attached to the main exhibition page and the exhibition was launched through an online event on November 12th as part of the national Being Human Festival [1]. Despite a thorough communications and press strategy, the intended reach of the exhibition was limited, with mainly academics and related professionals registering to watch the live launch. Local care-homes were understandably unresponsive to emails on the project given the COVID crisis, and all of the four public participants involved in the project who were over 80 could only be communicated with by phone and post, indicating a clear digital divide. Furthermore during the 10-day festival period, only one (albeit very positive) evaluation survey was completed, via a generic online festival evaluation survey that was directly linked to the online exhibition. Unfortunately there were also technical issues with the data collated via Google Analytics meaning this was highly limited. Any meaningful evaluation of the project was therefore made near impossible and despite some positive verbal feedback, it remains unclear as to what impact this project actually had. Our experience suggests that many of the older communities were unlikely to engage online at all due to either technical barriers, or the disproportionate effects of the pandemic on their care-home settings.

## Discussion

Case Studies One and Two were engagement events for adult audiences that took place in central Oxford prior to the pandemic. These events shared the common aim of encouraging public awareness and discussion of key ethical themes relevant to WEH research in UK healthcare. However, there were notable differences in the demographics of both audiences. The vaccination event attracted a much larger (n = 68) number of people than the Citizen Jury (n = 20), which was as expected given venue size, format of the event (an evening debate, versus a full day of lectures and discussion activities) and appeal of the topic. Furthermore, Case Study One was a partner event with a local festival that had pre-existing subscriptions and connections that had been established from previous work in broadening the reach of the festival to underserved audiences with lower science capital. As a result, a broader variety of views were apparent at the vaccination event, which in turn led to a much more varied and in-depth discussion of the issues, in comparison to our Citizen Jury where several attendees had previous knowledge on the topics given their voluntary roles in the local PPI network. Case Study Three, however, was instigated and coordinated during the pandemic and although featuring within a national festival, faced difficulties in reaching beyond an academic and professional audience.

Clearly opening up discussion to groups of publics was of benefit in Case Study One, as although views were more polarised, the discussion on vaccination was all the richer for it and misinformation, when it was raised, could be addressed. Views differed across the audience and even across the panel itself, but in being fully inclusive of all viewpoints and ensuring they were enabled to share a space, this event exposed the value in reaching beyond preconceived participants. On the contrary, Case Study Two struggled to broaden its reach to a wider range of views and although there was some disagreement in general discussions, the verdicts presented by the groups attending the Citizen Jury were all similar and shared some overlap, though this may also have been influenced by the subject matter. The intentions of Case Study Three were different, with the curation of an exhibition intended to provoke discussion, which may not have happened in the confines of the online event, but outcomes from Case Study Three were also harder to assess, due to the limited uptake of the evaluation online. Essentially inclusivity and reach was key for the success of Case Study One and the positive responses generated by evaluations on the event.

What interpretations can be made here on the effect of COVID-19 on engagement and would the outcomes of such events described here be affected if moved exclusively online, as a result of the pandemic? Humm and Schrögel [14] identify seven practical recommendations for engaging underserved groups, including listening to them, reducing the distance, going where the people are, cooperating with stakeholders and multipliers, illustrating the relevance of research for daily life, as well as opening up and making visible the research process, and creating sustainable, long-term activities.

By building on previous evaluation activities, WEH was able to learn about its audiences and participants, *listen* to them and build on engagement activities which work for them, but the relatively recent nature of the pandemic and the difficulties of pursuing engagement with evaluation online, as shown in Case Study Three, means there is still much to understand about what works for public engagement participants in online settings [20] and within the context of a pandemic [13, 23].

In terms of *distance*, WEH is no longer constrained to its local area and can now reach people in their own homes, around their commitments and synchronously or asynchronously. Oxford is somewhat unique in terms of its history and segregation of ‘Town and Gown’, but other UK universities, as well as those around the globe, will have similar challenges in seeking to play an active civic role in their local cities and communities, in which public engagement might also play a part. Our case studies suggest working with other *stakeholders and multipliers* relevant to local circumstances, people that can add to your efforts and may already have connections with those you seek to reach, perhaps becomes even more significant in identifying and appealing to a wider range of groups. Audience demographics are likely to be hindered by online only activities, as they require both technological ability, reliable internet access and capacity. Online engagement is ideal for ‘eye level’ dialogue [14] avoiding traditional hierarchies, but can raise questions of trust when webcams are turned on in people’s own homes, or activities must work around the constraints of shift work or home schooling. In many respects online engagement therefore extends the boundaries for ‘outreach’, which has been critiqued for further separating the excluded from the core business of institutions [17], and therefore in time it will also be important to continue to consider the implications this has for both institutional and personal spaces and settings.

By working with other stakeholders in Case Study One, targeted advertising and the enabling of key groups to participate was practical, however as community settings change and adapt in the social and economic echoes of the pandemic, so to may such relationships be challenged. Nonetheless the research of WEH remains highly relevant to *daily life*, perhaps even more so in the context of a pandemic when topics such as vaccines, health data and ethics have become day to day conversations amongst the media, as well as in many home and working contexts.

WEH have not run a panel debate or Citizen Jury online since the onset of the pandemic and most of WEH’s engagement work since March 2020 has been more informational, with less opportunity for two-way dialogue. However, COVID-19 has arguably brought into public view *research in progress* like never before, as understanding of the pandemic developed, data emerged and treatments and vaccines were sought, we have seen publics along the way influencing decision making, as well as public health messaging. As the pandemic continues to evolve, further thinking and reflection is required as to how the pandemic and resultant response measures have altered patient and public involvement in the research process [5, 13, 16, 23], as well as common engagement settings such as museum spaces [6].

A breadth of viewpoints better informs public awareness and enhances the quality and relevance of research, but ensuring this continues *sustainably* in purely online or blended engagement is a complex task that requires further long-term understanding and investment.

## Conclusions

Evidence from WEH’s pre-pandemic activities suggests that inclusivity in public engagement is an ongoing issue, where pre-engaged audiences are often repeatedly involved in events and activities, either through existing PPI networks or ineffective advertising. However, as evidence from the vaccination debate in Case Study One shows, audiences can often be reflective of broader groups with opposing views to those of the researchers or public majority, such as those identifying as vaccine hesitant or sceptic. This is particularly evident when the research presented is contentious or polarising.

But has the pandemic shifted mind-sets or perceived expectations around audiences and participants that might be reached and is it possible to tell? Does engagement need to adjust its long term goals, not only becoming more inclusive in its approach, but also becoming aware of and responsive to how and why these groupings adapt and shift over time? Moving engagement to a solely online format can create benefits in terms of inclusivity, for example in avoiding the need to travel costs, or mobility barriers for people with disabilities [19] and during the pandemic it played a vital role in protecting peoples’ health. But is also creates new barriers that must be further explored, if we are to fully understand how to broaden engagement reach in approaches such as that taken in Case Study Three. However, these are linked to pre-pandemic barriers to engagement [14], including ways to ensure public involvement is resilient, embedded and integral in research [5, 13, 23] albeit drawing out new connotations. Further exploration of these barriers is essential to both public awareness of and contribution to healthcare research, but also to ensuring that such research remains responsible, relevant and transparent, and that engagement takes place not only with a variety of people, but also with a diversity of viewpoints.

## Data Availability

‘Debating Data’ Citizens’ Jury report (Case Study Two): https://www.weh.ox.ac.uk/engagement. Accessed 23 June 2021.
